# A Nonlinear Calibration Algorithm Based on Harmonic Decomposition for Two-Axis Fluxgate Sensors

**DOI:** 10.3390/s18051659

**Published:** 2018-05-22

**Authors:** Wenguang Feng, Shibin Liu

**Affiliations:** School of Electronics and Information, Northwestern Polytechnical University, Xi’an 710072, China; fengwenguang@163.com

**Keywords:** fluxgate sensor, calibration algorithm, nonlinearity, harmonic decomposition

## Abstract

Nonlinearity is a prominent limitation to the calibration performance for two-axis fluxgate sensors. In this paper, a novel nonlinear calibration algorithm taking into account the nonlinearity of errors is proposed. In order to establish the nonlinear calibration model, the combined effort of all time-invariant errors is analyzed in detail, and then harmonic decomposition method is utilized to estimate the compensation coefficients. Meanwhile, the proposed nonlinear calibration algorithm is validated and compared with a classical calibration algorithm by experiments. The experimental results show that, after the nonlinear calibration, the maximum deviation of magnetic field magnitude is decreased from 1302 nT to 30 nT, which is smaller than 81 nT after the classical calibration. Furthermore, for the two-axis fluxgate sensor used as magnetic compass, the maximum error of heading is corrected from 1.86° to 0.07°, which is approximately 11% in contrast with 0.62° after the classical calibration. The results suggest an effective way to improve the calibration performance of two-axis fluxgate sensors.

## 1. Introduction

Two-axis fluxgate sensors are widely used as magnetometers or magnetic compasses, which determine the magnitude of magnetic fields or the heading of vehicles by measuring the Earth’s magnetic field in a horizontal plane, with widespread application to autonomous air, ground and ocean vehicles [1,2,3,4]. However, due to manufacturing technological limitations and magnetic interferences, the output of fluxgate sensors is corrupted by various errors, making it difficult to satisfy the precision requirement of practical applications [5]. Therefore, calibration of two-axis fluxgate sensors involving both identifying and compensating the errors is essential.

A well-known calibration procedure called compass swinging has been used successfully [6], which is based on the fact that the heading error is a Fourier function of the reference heading. The procedure involves rotating the vehicle through a series of reference headings, the differences between the raw heading measured by compass and the reference heading are used to compute calibration parameters. To simplify the compass swinging procedure, the angular-rate method using angular-rate information from low-cost micro-electro-mechanical system gyroscopes is presented in [7], which requires turning the compass through a full circle and only a single reference heading measurement, the difference of calibrated heading errors between these two methods is around 0.5°. In [8], the neural network algorithm is utilized to model the nonlinear mapping between the compass output and the reference heading. The calibration method is verified to be effective and robust, even in the presence of magnetic disturbances and large noises. The extreme learning machine algorithm is introduced to train a nonlinear error model in [9], of which the training speed is thousands of times faster than that of traditional back propagation neural network, the heading error is decreased from ±3° before calibration to ±0.2° after calibration. Nevertheless, all the methods mentioned above are only applicable for heading determination applications, and the quality of the calibration degrades when the vehicle with the fluxgate sensors moves far away from the geographical point where the calibration was performed, because the compensation coefficients are functions of the local magnetic field magnitude [10]. 

More calibration algorithms based on error models are presented, in which the error models are derived by analyzing the source of errors. The one-turn rotation scheme is to compensate for magnetic interference that changes the radius and shifts the center of the magnetism circle [11]. It uses the minimum and maximum value of each axis output to estimate the scale factor and bias errors, the shortcoming is that the algorithm performance is sensitive to noise. The batch least squares calibration algorithm in [12] accounts for the effects of hard iron, scale factor and part of soft iron. In this algorithm, a non-linear two-step estimator provides the initial conditions, which involves nonlinearly transforming compensation coefficients to ellipse parameters and fitting the best ellipse to the measured data, the obtained estimate of the compensation coefficients is then iteratively processed, the posterior covariance is used as a metric for the quality of the calibration. Furthermore, the batch least squares calibration algorithm is improved by compensating for the non-orthogonal error in [13]. The ellipse fitting problem in [12] is considered as a general conic fitting problem with a quadratic constraint, which is solved by using the Lagrange multiplier method [14]. A similar least squares ellipse fitting algorithm is presented in [15], three analytical manipulation methods transforming ellipse parameters to compensation coefficients are compared and discussed. However, there are at least two common drawbacks for these methods. One is that some kinds of errors are ignored, such as misalignment errors [16]. The other is that all error models are assumed to be linear models, and the nonlinearity of all errors is neglected. These drawbacks prominently limit the calibration performance. 

The nonlinearity of errors is influenced by many aspects. Cross-field effect and hysteresis are two important factors. The cross-field effect in perpendicular and transverse direction is described as an unwanted sensitivity or linearity error in [17], of which measurements are performed on fluxgate sensors with various constructions in [18], the results show that cross-field effect causes errors up to 40 nT in the earth’s magnetic field, and for miniature fluxgate sensor the error increases to 60 nT [19]. Several techniques for overcoming the cross-field effect have been presented in [20]. A nonlinear polynomial hysteresis model is proposed to analyze the influence on the sensitivity of sensor [21], and an actual hysteresis curve of a material is used for the theoretical analysis of the fluxgate sensor output [22]. Besides, nonlinear effects of the signal processing circuit and soft iron nearby are also worth of concern. A calibration algorithm with nonlinearity suppression is proposed in [23], which uses a third-order polynomial to parameterize the scale factor error, the magnetic field magnitude error is reduced by three times compared with that without nonlinearity suppression.

To improve the calibration performance for two-axis fluxgate sensors, a novel nonlinear calibration algorithm taking into account the nonlinearity of errors is proposed in this paper. The nonlinear error model is established by analyzing all time-invariant errors, harmonic decomposition method is presented for estimating the compensation coefficients. The performance of the proposed algorithm is experimentally validated and compared with a classical calibration algorithm. 

## 2. Error Modeling

In vehicle coordinate frame, the true value of magnetic field vector is denoted as $h_{t}$, the measured vector of two-axis fluxgate sensors is denoted as $h_{s}$. Due to the presence of errors, $h_{s}\neq h_{t}$. The errors should be equivalent parameterized, to establish the error model of two-axis fluxgate sensors. 

Error model is to describe the measured vector $h_{s}$ as a function of the true magnetic field vector $h_{t}$. In this paper, the function is divided into two parts: linear term and nonlinear term. Two vectors, ${\bar{h}}_{s}$ and ${\tilde{h}}_{s}$, are defined as the linear and nonlinear term about $h_{t}$, thus, $h_{s}$ can be written as:

(1) $$h_{s}={\bar{h}}_{s}+{\tilde{h}}_{s}$$

There are six typical error sources for two-axis fluxgate sensors, which are zero bias error $b_{\mathrm{zb}}$, scale factor error $S_{m}$, non-orthogonal error $C_{\mathrm{no}}$, soft iron error $C_{\mathrm{si}}$, hard iron error $b_{\mathrm{hi}}$, and misalignment error $C_{\mathrm{ma}}$ [24]. Each of these errors is discussed next in more detail. 

Zero bias error, also known as offset error, shifts the sensor output by a constant amount, which can be modeled as a 2 × 1 vector:

(2) $$b_{\mathrm{zb}}={\left[b_{\mathrm{zb}x}\;b_{\mathrm{zb}y}\right]}^{T}$$

where $b_{\mathrm{zb}x}$ and $b_{\mathrm{zb}y}$ are the non-zero value for the x-sensor and y-sensor of two-axis fluxgate sensors. 

Scale factor error is modeled using a 2 × 2 diagonal matrix $S_{m}$ given by:

(3) $$S_{m}=\left[\begin{array}{cc} s_{x} & 0\\ 0 & s_{y} \end{array}\right]$$

where $s_{x}$ and $s_{y}$ represent the constant of proportionality relating the output of fluxgate sensor to the true value of magnetic field component for each axis. Due to scale factor errors, $s_{x}\neq s_{y}$. In other words, when x-sensor and y-sensor of two-axis fluxgate sensors are subjected to an identical magnetic field, the output will not be the same. 

Non-orthogonal error comes from the nonorthogonality between x-sensor and y-sensor of two-axis fluxgate sensors. When x-sensor is assumed to be perfectly aligned with the *x*-axis in the vehicle coordinate frame, and an angle ρ is defined as being between the y-sensor and the *y*-axis [25], non-orthogonal error can be parameterized by a 2 × 2 lower triangular matrix:

(4) $$C_{\mathrm{no}}=\left[\begin{array}{cc} 1 & 0\\ \sin(\rho ) & \cos(\rho ) \end{array}\right]$$

Hard iron error is the result of unwanted magnetic fields generated by permanent magnets in the vicinity of sensors. It is constant in the vehicle coordinate frame, and can be represented as a 2 × 1 vector:

(5) $$b_{\mathrm{hi}}={\left[b_{\mathrm{hi}x}\;b_{\mathrm{hi}y}\right]}^{T}$$

where $b_{\mathrm{hi}x}$ and $b_{\mathrm{hi}y}$ are the null shift for each axis. 

Soft iron error is caused by the magnetization of soft magnetic materials, which generates their own field in response to the external magnetic field, the resulting magnetic field depends on the magnitude and direction of the applied external magnetic field. Here we assume that the relationship between the generated field and the externally applied field is linear and without hysteresis, soft iron error can be represented as a 2 × 2 matrix:

(6) $$C_{\mathrm{si}}=\left[\begin{array}{cc} c_{xx} & c_{xy}\\ c_{yx} & c_{yy} \end{array}\right]$$

The $c_{ij}$ terms represent the proportional constants between the magnetic field applied to soft magnets and the resulting magnetic field. For example, $c_{xy}$ represents the effective coefficient relating the resulting field generated in the *x*-axis direction in response to an applied field in the *y*-axis direction [12]. 

Misalignment error results from the misalignment between the individual sensors of the two-axis fluxgate sensor and the vehicle axes during installation. Referring to the vehicle coordinate frame, misalignment is equivalent to the rotation of a fluxgate sensor in a small angle $\varphi_{m}$, and the error can be expressed as a 2 × 2 orthogonal matrix:

(7) $$C_{\mathrm{ma}}=\left[\begin{array}{cc} \cos\varphi_{m} & -\sin\varphi_{m}\\ \sin\varphi_{m} & \cos\varphi_{m} \end{array}\right]$$

From the above, the linear term ${\bar{h}}_{s}$ in (1), as a linear function of the true magnetic field vector $h_{t}$, is described by:

(8) $$\begin{array}{ll} {\bar{h}}_{s} & =b_{\mathrm{zb}}+S_{m}C_{\mathrm{no}}(b_{\mathrm{hi}}+C_{\mathrm{si}}C_{\mathrm{ma}}h_{t})\\ & =b_{\mathrm{zb}}+S_{m}C_{\mathrm{no}}b_{\mathrm{hi}}+S_{m}C_{\mathrm{no}}C_{\mathrm{si}}C_{\mathrm{ma}}h_{t} \end{array}$$

Defining vectors $K_{e}=S_{m}C_{\mathrm{no}}C_{\mathrm{si}}C_{\mathrm{ma}}$ and $b_{e}=b_{\mathrm{zb}}+S_{m}C_{\mathrm{no}}b_{\mathrm{hi}}$, Equation (8) is transformed into

(9) $${\bar{h}}_{s}=b_{e}+K_{e}h_{t}$$

and the expanded form is:

(10) $$\left[\begin{array}{c} {\bar{h}}_{sx}\\ {\bar{h}}_{sy} \end{array}\right]=\left[\begin{array}{c} b_{e1}\\ b_{e2} \end{array}\right]+\left[\begin{array}{cc} k_{e11} & k_{e12}\\ k_{e21} & k_{e22} \end{array}\right]\left[\begin{array}{c} h_{tx}\\ h_{ty} \end{array}\right]$$

For error analysis of general sensors with nonlinearity, error model can usually be represented by the high order polynomial:

(11) $$y=\alpha_{0}^{}+\alpha_{1}x+\alpha_{2}x^{2}+\alpha_{3}x^{3}+\cdots$$

where y is the sensor output, x is the sensor input, and $a_{0}$, $a_{1}$, $a_{2}$, $a_{3}$, ⋯ are the polynomial coefficients. Here, regarding the nonlinearity of the scale factor error, soft iron error and cross-field effect of two-axis fluxgate sensors, this high order polynomial is used to parameterize the errors, then the error model (8) can be expanded in expansion form as:

(12) $$\left[\begin{array}{c} h_{sx}^{}\\ h_{sy}^{} \end{array}\right]=\left[\begin{array}{c} \alpha_{x0}+\alpha_{x1}h_{tx}^{}+\alpha_{x2}h_{ty}^{}+\alpha_{x3}{\left(h_{tx}^{}\right)}^{2}+\alpha_{x4}{\left(h_{ty}^{}\right)}^{2}+\alpha_{x5}h_{tx}^{}h_{ty}^{}+\cdots \\ \alpha_{y0}+\alpha_{y1}h_{tx}^{}+\alpha_{y2}h_{ty}^{}+\alpha_{y3}{\left(h_{tx}^{}\right)}^{2}+\alpha_{y4}{\left(h_{ty}^{}\right)}^{2}+\alpha_{y5}h_{tx}^{}h_{ty}^{}+\cdots \end{array}\right]$$

According to the definition of matrices in Equations (1) and (9), the nonlinear error model (12) including the nonlinearity of errors is rearranged in the matrix form as follows:

(13) $$\begin{array}{ll} h_{s}^{} & ={\bar{h}}_{s}+{\tilde{h}}_{s}\\ & =b_{e}+K_{e}h_{t}^{}+\xi_{e}^{}\nu_{t}^{} \end{array}$$

where $b_{e}^{}=\left[\begin{array}{c} \alpha_{x0}\\ \alpha_{y0} \end{array}\right]$, $K_{e}^{}=\left[\begin{array}{cc} \alpha_{x1} & \alpha_{x2}\\ \alpha_{y1} & \alpha_{y2} \end{array}\right]$, $\xi_{e}^{}=\left[\begin{array}{cccc} \alpha_{x3} & \alpha_{x4} & \alpha_{x5} & \cdots \\ \alpha_{y4} & \alpha_{y4} & \alpha_{y5} & \cdots \end{array}\right]$, $\nu_{t}^{}={\left[{\left(h_{tx}^{}\right)}^{2}\;{\left(h_{ty}^{}\right)}^{2}\;h_{tx}^{}h_{ty}^{}\;\cdots \right]}^{T}$. The expanded form is:

(14) $$\left[\begin{array}{c} h_{sx}^{}\\ h_{sy}^{} \end{array}\right]=\left[\begin{array}{c} b_{e1}\\ b_{e2} \end{array}\right]+\left[\begin{array}{cc} k_{e11} & k_{e12}\\ k_{e21} & k_{e22} \end{array}\right]\left[\begin{array}{c} h_{tx}^{}\\ h_{ty}^{} \end{array}\right]+\left[\begin{array}{c} \xi_{e11}{\left(h_{tx}^{}\right)}^{2}+\xi_{e12}{\left(h_{ty}^{}\right)}^{2}+\xi_{e13}h_{tx}^{}h_{ty}^{}+\cdots \\ \xi_{e21}{\left(h_{tx}^{}\right)}^{2}+\xi_{e22}{\left(h_{ty}^{}\right)}^{2}+\xi_{e23}h_{tx}^{}h_{ty}^{}+\cdots \end{array}\right]$$

## 3. Calibration Algorithm

Given an error model, calibration is the process that compensates the erroneous measured vector $h_{s}^{}$ to get the true magnetic field vector $h_{t}^{}$ by using the inverse function of the error model. Here, the inverse function of the error model is named as the calibration model. In the classical calibration algorithm, the nonlinear term in (1) is ignored, then the error model is expressed as:

(15) $$\begin{array}{ll} h_{s} & ={\bar{h}}_{s}\\ & =b_{e}+K_{e}h_{t} \end{array}$$

which is a linear error model, and the linear calibration model can be acquired directly from (15) through algebraic computation [26], yielding:

(16) $$h_{t}^{}=K_{c}(h_{s}^{}+b_{c})$$

where $K_{c}=K_{e}^{-1}$, $b_{c}=-b_{e}$. However, in the nonlinear calibration algorithm, as described in the nonlinear error model (14), the relationship between $h_{s}^{}$ and $h_{t}^{}$ is nonlinear, the inverse function of (14) cannot be obtained algebraically. 

**Figure 1:**
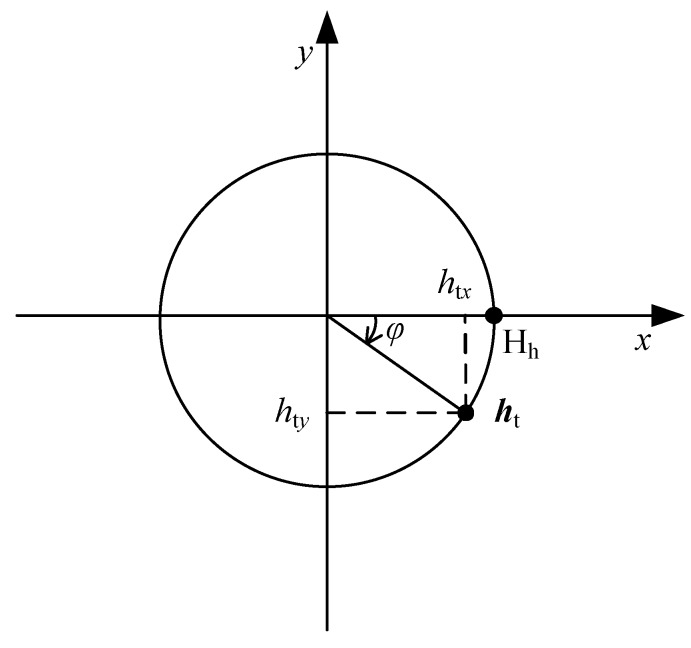
Relationship between the true value of magnetic field vector $h_{t}^{}$ and the reference heading φ.

In order to formulate the nonlinear calibration model, the characteristics of vector $h_{t}^{}$ and $h_{s}^{}$ are analyzed. While a two-axis fluxgate sensor is rotated in the horizontal plane, as shown in Figure 1, $h_{tx}^{}$ and $h_{ty}^{}$ can be expressed as functions of the reference heading φ by:

(17) $$\{\begin{array}{l} h_{tx}^{}(\varphi )=H_{h}\sin(\varphi )\\ h_{ty}^{}(\varphi )=H_{h}\cos(\varphi ) \end{array}$$

where x and y in subscripts indicate the corresponding axis direction in the vehicle coordinate frame, $H_{h}$ is the magnitude of the horizontal component of the earth’s magnetic field. $h_{sx}^{}$ and $h_{sy}^{}$ are also functions of φ, and satisfy:

(18) $$\{\begin{array}{l} h_{sx}^{}(\varphi )=h_{sx}^{}(2\pi i+\varphi )\\ h_{sy}^{}(\varphi )=h_{sy}^{}(2\pi i+\varphi ) \end{array}$$

where i=±1, ±2, ±3, ⋯.

As expressed in (18), $h_{sx}^{}(\varphi )$ and $h_{sy}^{}(\varphi )$ are periodic functions with a period of 2π, it is known that any periodic function satisfying the Dirichlet Condition can be expanded into the Fourier series in the form of trigonometric functions, thus $h_{sx}^{}(\varphi )$ and $h_{sy}^{}(\varphi )$ can be expressed as:

(19) $$\{\begin{array}{l} h_{sx}^{}(\varphi )=d_{0x}+\sum_{j=1}^{\infty }\left(d_{jx}\sin(j\varphi )+e_{jx}\cos(j\varphi )\right)\;(a)\\ h_{sx}^{}(\varphi )=d_{0y}+\sum_{j=1}^{\infty }\left(d_{jy}\sin(j\varphi )+e_{jy}\cos(j\varphi )\right)\;(b) \end{array}$$

Taking (a) in Equation (19) for example, it is expanded as

(20) $$h_{sx}^{}(\varphi )=d_{0x}+d_{1x}\sin(\varphi )+e_{1x}\cos(\varphi )+d_{2x}\sin(2\varphi )+e_{2x}\cos(2\varphi )+\cdots$$

On the right side of (20), there are constant term, fundamental terms and harmonic terms, thus, (20) is the harmonic decomposition of $h_{sx}^{}(\varphi )$. The properties of the Fourier series are known that the base functions are orthogonal to each other, and the coefficients are uncorrelated with each other. In conjunction with Equations (17) and (20) is transformed into:

(21) $$\begin{array}{ll} h_{sx}^{}(\varphi )= & \left(d_{0x}+e_{2x}+o_{0x}\right)\\ & +(\frac{d_{1x}}{H_{h}}+o_{1x}^{})h_{tx}^{}(\varphi )+(\frac{e_{1x}}{H_{h}}+o_{2x}^{})h_{ty}^{}(\varphi )\\ & +(\frac{2d_{2x}}{H_{h}{}^{2}}+o_{3x}^{})h_{tx}^{}(\varphi )h_{ty}^{}(\varphi )+(-\frac{2e_{2x}}{H_{h}{}^{2}}+o_{4x}^{}){\left(h_{tx}^{}(\varphi )\right)}^{2}+\cdots \end{array}$$

where $o_{0x}$, $o_{1x}$, $o_{2x}$, $o_{3x}$ and $o_{4x}$ are the coefficient sums of constant, $h_{tx}^{}(\varphi )$, $h_{ty}^{}(\varphi )$, $h_{tx}^{}(\varphi )h_{ty}^{}(\varphi )$ and ${\left(h_{tx}^{}(\varphi )\right)}^{2}$ terms in expansion of the third and higher harmonic terms, respectively. The simplified form of (21) is expressed as:

(22) $$\begin{array}{ll} h_{sx}^{}= & \left(d_{0x}+e_{2x}+o_{0x}\right)\\ & +(\frac{d_{1x}}{H_{h}}+o_{1x}^{})h_{tx}^{}+(\frac{e_{1x}}{H_{h}}+o_{2x}^{})h_{ty}^{}\\ & +(\frac{2d_{2x}}{H_{h}{}^{2}}+o_{3x}^{})h_{tx}^{}h_{ty}^{}+(-\frac{2e_{2x}}{H_{h}{}^{2}}+o_{4x}^{}){\left(h_{tx}^{}\right)}^{2}+\cdots \end{array}$$

Given the same expansion process (20)–(22) for (b) in Equation (19), $h_{sy}^{}$ is expressed as:

(23) $$\begin{array}{ll} h_{sy}^{}= & \left(d_{0y}-e_{2y}+o_{0y}\right)\\ & +(\frac{d_{1y}}{H_{h}}+o_{1y}^{})h_{tx}^{}+(\frac{e_{1y}}{H_{h}}+o_{2y}^{})h_{ty}^{}\\ & +(\frac{2d_{2y}}{H_{h}{}^{2}}+o_{3y}^{})h_{tx}^{}h_{ty}^{}+(\frac{2e_{2y}}{H_{h}{}^{2}}+o_{4y}^{}){\left(h_{ty}^{}\right)}^{2}+\cdots \end{array}$$

The two expressions above can be rewritten into the matrix form as the nonlinear error model (13):

(24) $$h_{s}^{}=b_{e}+K_{e}h_{t}^{}+\xi_{e}^{}\nu_{t}^{}$$

where $b_{e}^{}=\left[\begin{array}{c} d_{0x}+e_{2x}+o_{0x}\\ d_{0y}-e_{2y}+o_{0y} \end{array}\right]$, $K_{e}^{}=\left[\begin{array}{cc} \frac{d_{1x}}{H_{h}}+o_{1x} & \frac{e_{1x}}{H_{h}}+o_{2x}\\ \frac{d_{1y}}{H_{h}}+o_{1y} & \frac{e_{1y}}{H_{h}}+o_{2y} \end{array}\right]$, $\xi_{e}^{}=\left[\begin{array}{cccc} -\frac{2e_{2x}}{H_{h}{}^{2}}+o_{4x} & 0 & \frac{2d_{2x}}{H_{h}{}^{2}}+o_{3x} & \cdots \\ 0 & \frac{2e_{2y}}{H_{h}{}^{2}}+o_{4y} & \frac{2d_{2y}}{H_{h}{}^{2}}+o_{3y} & \cdots \end{array}\right]$, $\nu_{t}^{}={\left[\begin{array}{cccc} {\left(h_{tx}^{}\right)}^{2} & {\left(h_{ty}^{}\right)}^{2} & h_{tx}^{}h_{ty}^{} & \cdots \end{array}\right]}^{T}$. Compared with expression (13), it should be noted that some elements in matrix $\xi_{e}$ of expression (24) are zero, which means that only a part of high order terms in (13) is sufficient to describe the nonlinear error model. 

From Equation (24), $h_{t}^{}$ is given by:

(25) $$h_{t}^{}=K_{c}(b_{c}+h_{s}^{})+\xi_{c}^{}\nu_{t}^{}$$

where $K_{c}=K_{e}^{-1}$, $b_{c}=-b_{e}$, $\xi_{c}=-K_{e}^{-1}\xi_{e}$. Defining ${\bar{h}}_{t}^{}=K_{c}(b_{c}+h_{s}^{})$, (25) is rewritten as:

(26) $$h_{t}^{}={\bar{h}}_{t}^{}+\xi_{c}^{}\nu_{t}^{}$$

$\nu_{t}^{}$ is composed of the high-order terms about $h_{t}^{}$, which comes from the harmonic terms in (20). As the total linearity of fluxgate sensors is relatively good, coefficients of the harmonic terms in (20) are much smaller than that of the fundamental terms, i.e., for j≥2, there are $d_{j}\ll d_{1}$ and $e_{j}\ll e_{1}$. Thus, $h_{t}^{}$ in $\nu_{t}^{}$ can be replace with ${\bar{h}}_{t}^{}$, and (26) is transformed into:

(27) $$\begin{array}{ll} h_{t}^{} & \approx {\bar{h}}_{t}^{}+\xi_{c}^{}{\bar{\nu }}_{t}^{}\\ & ={\bar{h}}_{t}^{}+{\tilde{h}}_{t}^{} \end{array}$$

where ${\bar{\nu }}_{t}^{}={\left[{\left({\bar{h}}_{tx}^{}\right)}^{2}\;{\left({\bar{h}}_{ty}^{}\right)}^{2}\;{\bar{h}}_{tx}^{}{\bar{h}}_{ty}^{}\;\cdots \;\right]}^{T}$. Equation (27) is the approximate expression of the nonlinear calibration model, and the expansion form is given by:

(28) $$\left[\begin{array}{c} h_{tx}^{}\\ h_{ty}^{} \end{array}\right]=\left[\begin{array}{c} {\bar{h}}_{tx}^{}+\xi_{c11}^{}{\left({\bar{h}}_{tx}^{}\right)}^{2}+\xi_{c12}^{}{\bar{h}}_{tx}^{}{\bar{h}}_{ty}^{}+\cdots \\ {\bar{h}}_{ty}^{}+\xi_{c21}^{}{\left({\bar{h}}_{ty}^{}\right)}^{2}+\xi_{c22}^{}{\bar{h}}_{tx}^{}{\bar{h}}_{ty}^{}+\cdots \end{array}\right]$$

where $\left[\begin{array}{c} {\bar{h}}_{tx}^{}\\ {\bar{h}}_{ty}^{} \end{array}\right]=\left[\begin{array}{cc} k_{c11}^{} & k_{c12}\\ k_{c21}^{} & k_{c22}^{} \end{array}\right]\left(\left[\begin{array}{c} b_{c1}^{}\\ b_{c2}^{} \end{array}\right]+\left[\begin{array}{c} h_{sx}^{}\\ h_{sy}^{} \end{array}\right]\right)$. 

To complete the nonlinear calibration algorithm, compensation coefficients in (28) should be determined. According to the relationship between Equations (20) and (24), it is known that the error coefficients $b_{e}$, $K_{e}$ and $\xi_{e}$ are weak correlated with each other. Thus, the determination of compensation coefficient can be performed iteratively, and in each iteration the linear compensation coefficients $b_{c}$ and $K_{c}$, the nonlinear compensation coefficients $\xi_{c}$ are estimated respectively. The iterative steps are as follows:

1. For N raw data collected from the two-axis fluxgate sensor, set the highest order of the harmonic terms in (19), denoted by J, the initial value ${\tilde{h}}_{t}{}^{\left(0\right)}{=[0\;0]}^{T}$, and the number of iterations i=0. Define the error of magnetic field $\delta h_{t}^{}{}^{\left(0\right)}=h_{t}^{}$.
2. Calculate the linear compensation coefficients $K_{c}{}^{\left(i\right)}$ and $b_{c}{}^{\left(i\right)}$ by solving the linear equation:
  (29) $$\delta h_{t}{}^{\left(i\right)}=K_{c}{}^{\left(i\right)}\left(b_{c}{}^{\left(i\right)}+h_{s}^{}\right)$$
3. Calculate the nonlinear compensation coefficients $\xi_{c}{}^{\left(i\right)}$ by solving the linear equation:
  (30) $$\delta h_{t}^{}{}^{\left(i\right)}-{\bar{h}}_{t}^{}{}^{\left(i\right)}=\xi_{c}^{}{}^{\left(i\right)}\;{\bar{\nu }}_{t}^{}{}^{\left(i\right)}$$
4. Calculate the error of magnetic field by:
  (31) $$\delta h_{t}^{}{}^{\left(i+1\right)}=\delta h_{t}^{}{}^{\left(i\right)}-({\bar{h}}_{t}{}^{\left(i\right)}+{\tilde{h}}_{t}{}^{\left(i\right)})$$
  and the root mean square (RMS) error of magnetic field by:
  (32) $$\sigma =\sqrt{\frac{1}{N-1}\sum_{n=1}^{N}{\left(\delta h_{t}^{}(n)\right)}^{2}}$$
  where n=1, 2, ⋯, N. Repeat step 1 to step 3 until σ reaches a predefined threshold or the minimum value.

In the iteration, the highest order of the harmonic terms J is limited by the number of raw data N. In practice, more raw data are usually collected to reduce the influence of random errors and improve the accuracy of calibration, and the least square method is used to solve Equations (29) and (30).

After the iteration, the true magnetic field vector $h_{t}^{}$ can be calculated by:

(33) $$h_{t}^{}=\sum_{i=1}^{I}\left[K_{c}{}^{\left(i\right)}(b_{c}{}^{\left(i\right)}+h_{s}^{})+\xi_{c}^{}{}^{\left(i\right)}\nu_{t}^{}{}^{\left(i\right)}\right]$$

where I is the maximum number of iterations.

## 4. Experiments and Discussions

**Figure 2:**
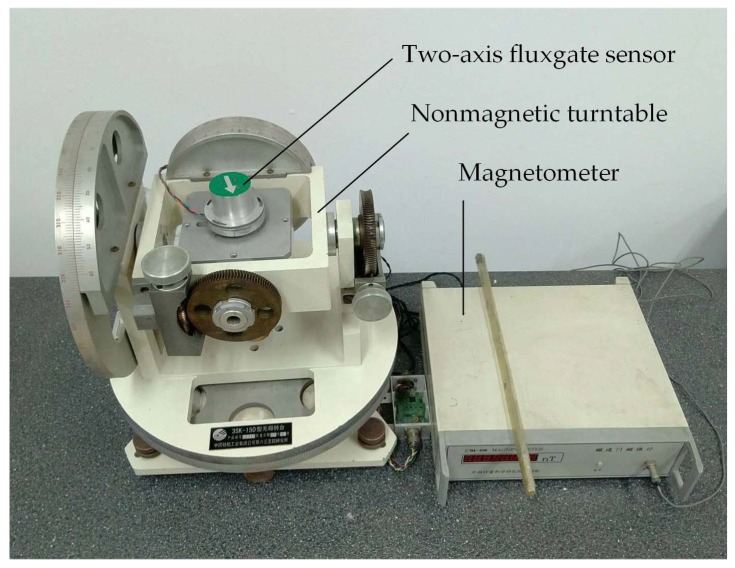
Experimental platform.

The proposed nonlinear calibration algorithm is verified by experiments, and compared with a classical calibration algorithm which is based on the linear error model (15) mentioned earlier. The experiment is performed in the laboratory, and the Earth’s magnetic field is used for measurement. The experimental platform, as shown in Figure 2, consists of a CTM-6W magnetometer, a two-axis fluxgate sensor (to be calibrated) [27], and a 3SK-150 nonmagnetic turntable. The CTM-6W magnetometer, with accuracy of ±1 nT, is used to measure the true value of magnetic field magnitude. The 3SK-150 nonmagnetic turntable, of which the heading accuracy is less than 0.05°, is adopted to provide the reference heading for the two-axis fluxgate sensor. The two-axis fluxgate sensor is rigidly mounted onto the nonmagnetic turntable to obtain experimental data with different headings. 

**Figure 3:**
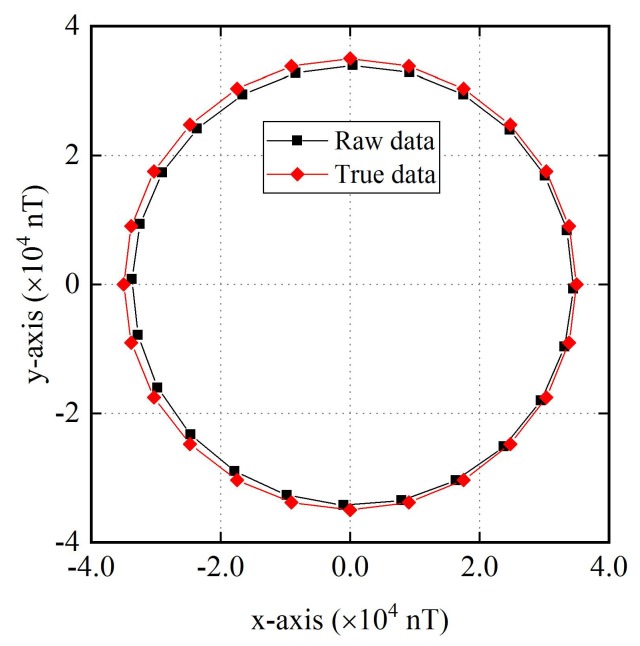
Raw data of the two-axis fluxgate sensor and true data.

In the experiment, the turntable is rotated in one circle, and raw data of the two-axis fluxgate sensor are collected with heading interval of 15°. Meanwhile, the true data at each point is calculated using Equation (17). The total 24 sets of data are displayed in Figure 3. 

**Figure 4:**
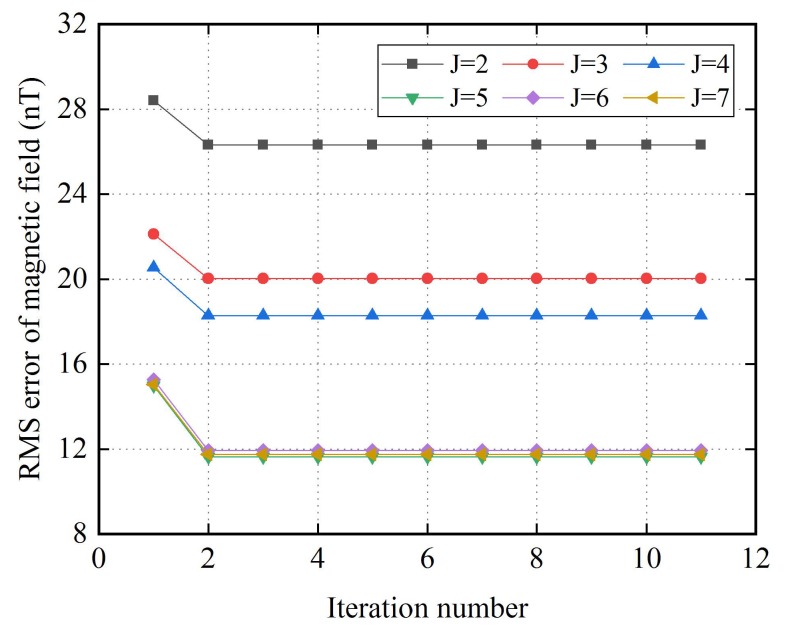
The RMS errors of magnetic field after each iteration when the highest order of harmonic terms J is set from 2 to 7.

Using the data set in Figure 3, the performance of the proposed nonlinear calibration algorithm is evaluated. Before calibration, the RMS errors of magnetic field is 920.0 nT. After calibration, while the highest order of harmonic terms J is set to 2, 3, 4, 5, 6 and 7, the RMS error of magnetic field is reduced to about 26.3 nT, 20.0 nT, 18.3 nT, 11.6 nT, 11.9 nT and 11.7 nT, respectively. The detailed calibration processes are illustrated in Figure 4. As can be seen, the nonlinear calibration algorithm converges fast, and after 2 iterations, the RMS errors of magnetic field nearly reach the minimum value, this is mainly because that the correlation between error coefficients in (24) is weak. With the increase of the highest order value of harmonic terms J, the RMS error of magnetic field decreases. When the highest order of harmonic terms J is large than 5, there is no significant difference between the RMS errors of magnetic field, which means that harmonic errors with orders no greater than 5 are the main components of the error. Although the errors are reduced by tens of times, there are residual errors after calibration, this is mainly because that hysteresis, temperature error and random noise have not been completely solved. In addition, due to the digital output of the two-axis fluxgate sensor, quantization noise is also an important factor. 

**Figure 5:**
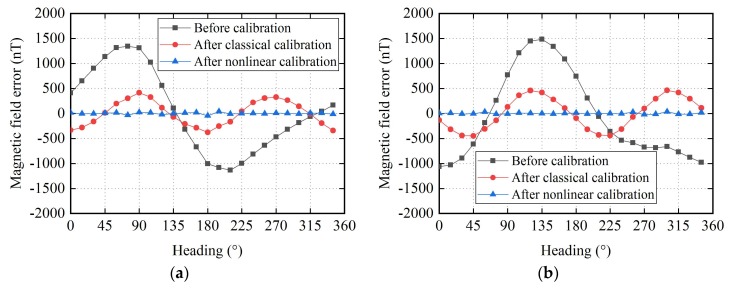
Errors of the magnetic field component for each axis before and after calibration: (**a**) *x*-axis; (**b**) *y*-axis.

**Figure 6:**
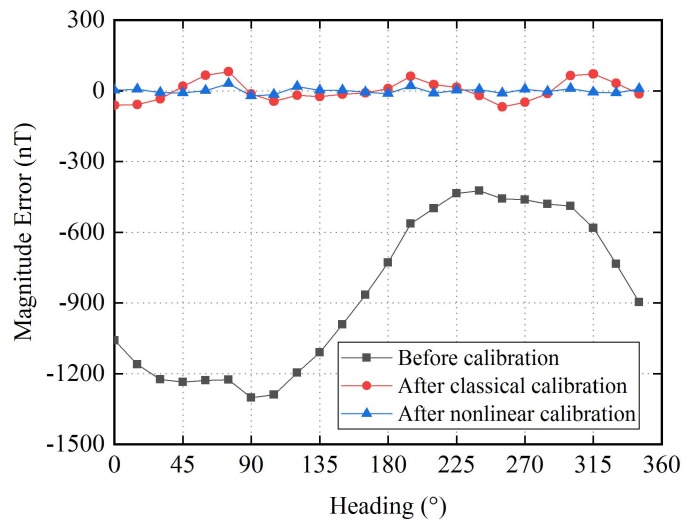
Errors of the magnetic field magnitude before and after calibration.

**Figure 7:**
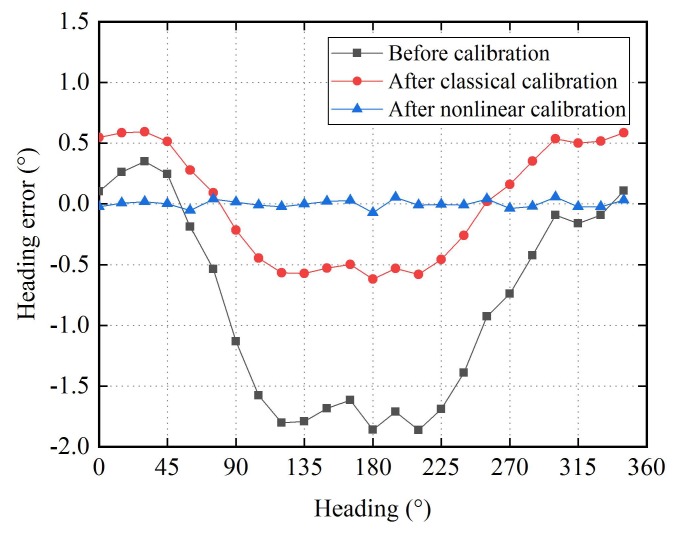
Heading errors before and after calibration.

Comparison of performance between the proposed nonlinear calibration algorithm and the classical calibration algorithm is performed. Here, the highest order of harmonic terms J is set to 5. The calibration results are illustrated in Figure 5, Figure 6 and Figure 7. Figure 5 shows the error of the magnetic field component for each axis before and after calibration. It is obvious that, in the raw data there are harmonic errors, especially the fundamental terms, in the classical calibrated data the fundamental errors have been eliminated and the second harmonic terms are the main components of errors, in the nonlinear calibrated data there are no significant harmonic errors. Figure 6 shows the error of magnetic field magnitude. Before calibration. There are an offset of about 860.4 nT and harmonic errors, peculiarly the fundamental term. After classical calibration, the offset and the fundamental error are compensated, and the third harmonic term becomes the major component of error. After nonlinear calibration, all the harmonic errors are well compensated. Figure 7 shows the heading error when the two-axis fluxgate sensor is use as magnetic compass. Before calibration, an offset of about 0.84° and fundamental term are the main part of error. After classical calibration, the offset are eliminated, and the fundamental error is significantly reduced, which is still the major part of error. After nonlinear calibration, the harmonic errors are well corrected. 

From Figure 5, Figure 6 and Figure 7, it can be seen that the offset and the harmonic errors are prominent before calibration, only the offset and the fundamental errors can be partially compensated with the classical calibration algorithm, and all the harmonic errors with orders no greater than 5 are effectively compensated with the nonlinear calibration algorithm. The maximum value of the errors in these figures are listed in Table 1.

In a word, the experimental results show that the proposed nonlinear calibration algorithm is more effective than the classical calibration algorithm, and it should be a trade-off between computational complexity and calibration accuracy for the nonlinear calibration algorithm. 

## 5. Conclusions

A novel nonlinear calibration algorithm is proposed and successfully validated for calibrating two-axis fluxgate sensors. The combined effort of all time-invariant errors are analyzed to establish the nonlinear error model, and harmonic decomposition method is presented for estimating the compensation coefficients. The performance of the proposed nonlinear calibration algorithm is compared with that of a classical calibration algorithm by performing experiments on a nonmagnetic turntable, the experimental results show that after the nonlinear calibration, the maximum deviation of magnetic field magnitude is decreased from 1302 nT to 30 nT, which is smaller than 81 nT after the classical calibration. Furthermore, for the two-axis fluxgate sensor used as magnetic compass, the maximum error of heading is corrected from 1.86° to 0.07°, which is approximately 11% in contrast with 0.62° after the classical calibration. The proposed algorithm can be effectively applied to calibrate any two-axis sensor. Future work will exploit the adaptation of this algorithm to three-axis sensors. 

## Figures and Tables

**Table 1:** The maximum error before and after calibration.

	*x*-axis Magnetic Field (nT)	*y*-axis Magnetic Field (nT)	Magnetic Field Magnitude (nT)	Heading (°)
Before calibration	1345	1483	1302	1.86
After classical calibration	419	464	81	0.62
After nonlinear calibration	36	34	30	0.07
